# Long-Term Results of Choledochoduodenostomy

**DOI:** 10.1155/1992/20567

**Published:** 1992

**Authors:** J. P. Neoptolemos, S. Radley

**Affiliations:** University Department of Surgery Dudley Road Hospital Birmingham B18 7QH UK


*HPB Surgery,* 1992, Vol. 5, pp. 157–159
Reprints available directly from the publisher
Photocopying permitted by license only

© Harwood Academic Publishers GmbH
Printed in the United Kingdom

**HPB INTERNATIONAL**

**EDITORIAL & ABSTRACTING SERVICE**

JOHN TERBLANCHE, EDITOR
Department of Surgery,
Medical School · Observatory 7925
Cape Town · South Africa
Telephone: (021) 47-1250 Ext. 2
Telefax (021) 448-6461

### LONG-TERM RESULTS OF CHOLEDOCHODUODENOSTOMY

ABSTRACT

*Escudero-Fabre, A., Escallon, A., Sack, J., Halpern, N.B., Aldrete, J.S. (1991) Cholodechoduodenostomy. Analysis of 71 cases followed for 5 to 15 years. Annuals of Surgery;* **213**: 635–644.

**To investigate the long-term effectiveness of choledochoduodenostomy (CDD), the experience with 71 patients followed for 5 or more years after CDD was analyzed retrospectively. From 1968 to 1984, 134 patients underwent CDD. Eight patients (6%) died in the immeditate postoperative period, 55 left the hospital, 8 of them were lost to follow-up, and 47 were followed but died before 5 years elapsed after CDD. The remaining 71 patients form the date base for this analysis: 38 were followed for more than 5 years, 25 were followed for more than 10 years and 8 were followed for more than 15 years ($\bar{x}$ 12.1 years ± 1.3 SEM). Choledocholithiasis, chronic pancreatitis and postoperative stricture were the indications for CDD. Cholangitis was observed in only three patients. The diameter of the common bile duct (CBD) was large in most patients ($\bar{x}$ 18 mm ± 0.9 SEM). These results infer that CDD is effective to treat non-neoplastic obstructing lesions of the distal CBD on a long-term basis and that the presence of a dilated CBD (more than 16 mm) that allows the construction of a CDD more than 14 mm is essential to obtain good results.**

### PAPER DISCUSSION

KEY WORDS: Choledocholithiasis, choledochoduodenostomy

The role of biliary tract surgery has come under considerable scrutiny since the advent of therapeutic endoscopic retrograde cholangiopancreatography (ERCP) and more recently that of laparoscopic cholecystectomy. One of the arguments that could be effectively used against the routine use of endoscopic sphinctermotomy (ES) and bile duct stone extraction was the 10–20% risk to the patients of developing subsequent gallbladder symptoms including chronic cholecystitis and empyema of the gall bladder [1]. On the existing evidence it would appear that a combination of ES and laparoscopic cholecystectomy[2] would be preferable to open surgery. Laparoscopic choledocholithotomy can be performed but the success rate is likely to be higher with ES, a procedure associated with a low morbidity and mortality [1]. Although this should not be completely pre-judged, particularly since a prospective trial failed to show a superior advantage of ES combined with conventional cholecystectomy over surgery alone [3], the concept of minimally-invasive surgery is likely to be preferred by patients.

In deciding treatment options both the immediate post-procedural and long-term complications need to be compared. Whereas there is reasonably good comparative data for the former, good long-term results are relatively lacking both with regards to surgical choledocholithotomy and to endoscopic treatment. These arguments apply equally to the definitive treatment of bile duct stones by percutaneous techniques [4]. Not withstanding the lack of any long-term data, percutaneous methods are likely to be inferior given the relatively poor initial success rates and high complication rates; such arguments may not apply in the context of recurrent pyogenic cholangitis and intrahepatic duct calculi.

This contribution of Escudero-Fabre and colleagues contains data of considerable importance to these arguments. This is an enjoyable paper, well written and excellently referenced. The series is, however, slightly confused by including a number of patients known to have had cancer at the time of initial surgery. It is preferable to exclude these and consider only these 104 patients operated on for presumed benign disease. In this group, there were three (2.9%) post-operative deaths; eight patients were lost to follow-up; thirteen died up to 5 years from unrelated causes; nine died of malignancy (in six cases due to pancreatico-biliary cancer). There remained 71 patients who were followed up for 5–15 years (mean 12.1 years) of whom three (4.2%) developed attacks of acute cholangitis; one had one attack managed conservatively, another had multiple attacks until a metal stent was inserted percutaneously and a third died from liver failure as a consequence of (primary) sclerosing cholangitis.

The results confirm the relatively low mortality of this operation. Originally this was performed by Sprengel [9] in 1891 and not by Riedel in 1888 as is so often quoted. It was extensively used with considerable success by German surgeons but curiously it was subsequently rejected by them because of the fear of recurrent cholangitis but detailed evidence of this was lacking. The overall incidence of long-term complications reported by Escudero-Fabre *et al.* is consistent with several studies involving detailed long-term follow-up of choledochoduodenostomy all showing an incidence of sump syndrome and/or cholangitis of $<5\%$ [7,8,10]. Most of these complications are readily dealt with by endoscopic treatment [11,12]. The data on post-procedural mortality and long-term results are similar to those reported for endoscopic sphincterotomy as the primary treatment [1].

Of particular concern was the finding that six (5.7%) patients had pancreaticobiliary tumours unsuspected at the time of choledochoduodenostomy. Thomas *et al.* [13] reported a similar incidence of 5% in 1971 and Baker *et al.* [7] had an incidence of 3.7%. Since these tumours could not be readily differentiated from the oedema in the pancreatic head caused by impacted gallstones, it suggests that they were all resectable. This further supports the argument for detailed pre-operative investigation in all patients presenting with obstructive jaundice — preferably by ERCP.

Choledochoduodenostyomy has a definite role in the management of bile duct stones. The proportion of cases requiring this approach is diminishing because of non-operative techniques but it will not be eliminated by them based on current trends.

### REFERENCES

1. Winslet, M.C., Neoptolemos, J.P. (1991) The place of endoscopy in the management of gallstone. *Clinical Gastroenterology*, **5**, 99–129
2. Southern Surgeon's Club. (1981) A prospective analysis of 1518 laparoscopic cholecystectomies. *N. Eng. J. Med.*, **324**, 1073–1077
3. Neoptolemos, J.P., Carr-Locke, D.L., Fossard, D.P.(1987) Prospective randomised study of preoperative endoscopic sphincterotomy versus surgery alone for common bile duct stones. *Br. Med. J.*, **294**, 470–474
4. Stokes, K.R., Falchuk, K. R., Clouse, M.E. (1989) Biliary duct stones:updated on 54 cases after percutaneoustrashepaticremoval. *Radiology*, **170**, 999–1001
5. Madden, J.L., Chun, J. Y., Kandalaft, S., Parakh, M. (1970) Choledochoduodenostomy. An unjustly maligned surgical procedure? *Am. J. Surg.*, **119**, 45–54
6. Schein, C.J., Gliedman, M.L. (1981) Choledochoduodenostomy as an adjunt to choledocholithotomy. *Surg. Gynecol. Obstet.*, **152**, 797–804
7. Baker, A.R., Neoptolemos, J.P., Leese, T., James, D.C., Fossard, D.P.(1987)Longtermfollow-upofpatientswithsidetoside choledochoduodenostomy and transduodenal sphincteroplasty. *Ann. Roy. Coll. Surg. Eng.*, **68**, 253–257
8. Parpilla, P., Ramirex, P., Sanchez Bueno, F., Perex, J.M., Candel,M.F.,Muelas, M.S., Robles, R. (1991) Longtermresultsof choledochoduodenostomy in the treatment of choledocholithiasis: assessment of 225 cases. *Br. J. Surg.* **78**, 470–472
9. Sprengel, O. (1891) Uber einen Fall Von Extripation der Gallenblasse mit Anlegung einer Communication Zwischen Ductus Choledochus und Duodenum. *Arch. Klin, Chir.* **156**, 417
10. Hughier, M., Lacaine, F., Aoury, S., Pascal, G. (1985) Choledochodenostomy for calculous biliary tract disease. *Arch. Surg.* **147**, 253–259
11. Barkin, J.S., Silvis, S., Greenwald, R. (1980) Endoscopic therapy of the 'sump' syndrome. *Dig. Dis. Scit.*, **25**, 597–601
12. Baker, A,R., Neoptolemos, J.P., Carr-Locke, D.L., Fossard, D.P. (1985) Sump syndrome following choledochoduodenostomy and its endoscopic treatment. *Br. J. Surg.*, **72**, 433–435
13. Thomas, C.G., Nicholson, C.P., Owen, J.(1971) Effectiveness of choledochodueodenostomy and transduodenal sphincterotomy in the treatment of benign obstruction of the common duct. *Ann. Surg.*, **173**, 845–856

J.P. Neoptolemos
Reader in Surgery
S. Radley
Cancer Research Campaign, Research Fellow
University Department of Surgery
Dudley Road Hospital
Birmingham B18 7QH
United Kingdom

